# Editorial: Death Receptors, Non-apoptotic Signaling Pathways and Inflammation

**DOI:** 10.3389/fimmu.2020.02162

**Published:** 2020-09-08

**Authors:** Eva Szegezdi, Patrick Legembre

**Affiliations:** ^1^Discipline of Biochemistry, National University of Ireland, Galway, Ireland; ^2^INSERM U1262, CRIBL, Université Limoges, Limoges, France

**Keywords:** TNF, apoptosis, inflammation, death receptor, nuclear factor kappa B

The tumor necrosis factor (TNF) receptor family encompasses a death receptor (DR) sub-group. While DRs are known for their ability to trigger cell death responsible for the elimination of transformed or infected cells and the homeostasis of the immune system, they also induce non-apoptotic signaling promoting proliferation, differentiation, and migration of various cells contributing to organ development and to inflammation (1, 2). While a large body of research has linked deregulation of death receptor-mediated cell death signaling to progression of human diseases, such as chronic inflammatory disorders and cancers (3), recent studies have revealed that non-apoptotic signaling pathways driven by death receptors could also interfere with the etiology of these disorders.

CD95 (also known as Fas), TNF-related apoptosis-inducing ligand (TRAIL) receptors (DR4/TRAILR1 and DR5/TRAILR2), TNFR1, DR3, DR6, nerve growth factor receptor (NGFR), and ectodysplasin receptor (EDAR) are members of the TNF DR sub-family. A common feature of these receptors is the presence of a distinctive intracellular domain, designated as the death domain (DD), which is responsible for the initiation of apoptotic signal transduction. Upon activation of a DR, the DD recruits the adaptor protein FADD on which the molecular platform responsible for the initiation of apoptosis assembles.

While the core molecular events triggering cell death upon DR activation is well-understood, the signal transduction pathways driving non-apoptotic signaling are far less resolved. Indeed, although the initial events leading to the induction of nuclear factor kappa B (NFκB), mitogen-activated protein kinase (MAPK) or cell death by TNFR1-engagement has been widely studied (4), the molecular mechanisms initiating non-apoptotic signaling pathways by CD95, DR4, and DR5 remains elusive and would benefit from further investigation in the future to (i) address how apoptotic and non-apoptotic machineries are interconnected and thereby controlled and (ii) understand the pathophysiological roles of these non-apoptotic signaling pathways and finally (iii) to develop novel therapeutics selectively targeting these pathways.

By recruiting different factors including adaptors (e.g., FADD, TRADD), caspases (caspase-8 and −10), phosphatases, [e.g., Fas-associated phosphatase-1 (5)], and kinases [e.g., receptor interacting kinase (RIP)-1, RIP3], death receptors assemble a dynamic and adaptive molecular platform leading to the simultaneous and/or sequential ignition of various signals. This complexity makes deciphering the pathological role of each individual death receptor-triggered signal very challenging in both cancers and auto-immune disorders. In addition to this molecular complexity, the microenvironment, meaning the tumor- or the inflamed tissues, also modulate death receptor signaling via secretion of death ligands, mechanical stress, altered local pH (e.g., acidity), hypoxia, and glucose concentration in a fashion that can also affect how death receptors respond and render the biological outcome of agonist or antagonist therapeutics difficult to predict (de Looff et al.). With our increasing understanding and recognition of the importance of non-apoptotic functions of death receptors, their role in the progression of inflammatory pathologies, cancers and graft-*versus*-host disease has to be re-assessed.

Inflammation is a protective attempt by the host to remove injurious stimuli and initiate the tissue healing process. While inflammation is essential for tissue repair, failure to end the inflammatory response leads to off-target damage in tissues and the development of chronic inflammatory and auto-immune diseases. This special issue presents recent developments and novel methodologies of research in this field. For instance, NFκB activation induced by CD95 is required to prime the inflammasome and thereby to secrete the pro-inflammatory cytokines interleukin (IL)-1β and IL-18. A novel tool using multispectral imaging flow cytometry is reported in this special issue to study the activation of this complex (Lage et al.).

Death receptor signaling is also increasingly recognized to shape the interactions between tumor cells and the immune complement in the tumor microenvironment (de Looff et al.). For example, our increasing understanding of the multitude of signal transduction pathways triggered by TNF receptors (6) sheds light to reasons for the positive as well as negative effects of TNF in tumor progression (Montfort et al.). Also, with our increasing understanding of the non-apoptotic responses triggered by death receptors, it emerges that DRs have a much deeper and widespread impact on the immune response than previously assumed, from controlling the expansion of regulatory T cells (Treg) by DR3 (Mavers et al.), the role of TRAIL in activation induced cell death of B cells (Staniek et al.) and CD95-mediated stimulation of pro-inflammatory Th17 cell trafficking in damaged kidneys of lupus patients (7) to the possible role of TRAIL decoy receptor 2 (DcR2/TRAIL-R4) in certain cancer enhancing the anti-tumor activity of γδ T lymphocytes by downregulating cyclooxygenase (COX)-1 and COX-2 expressions and consequent prostaglandin E2 (PGE2) secretion (Tawfik et al.). Besides, soluble TNF (Montfort et al.) and CD95L (8) share the ability to promote epithelial-to-mesenchymal transition (EMT) [Montfort et al.; (9)], a pivotal process aiding wound healing but also a molecular mechanism involved in metastatic dissemination in cancer.

Finally, our view and understanding on the mechanism of cell death induced by death receptors has also evolved with recent studies highlighting that DR activation can trigger not only apoptosis, but also other forms of regulated cell death, including necroptosis and pyroptosis and the molecular links existing between these different types of cell death emphasize that inhibition of apoptosis rather than resulting in cell survival, may activate a secondary or a tertiary one to avoid survival of infected and transformed cell (10). Of note, cell death occurring through pyroptosis and necroptosis have been reported to stimulate the immune response, while apoptosis is still considered tolerogenic (11). Thus, the interplay between death receptor-mediated cell death and inflammation will require further investigation to decipher whether the combination of a given type of cell death with an inflammatory program dictates whether an immunogenic or tolerogenic cell death pathway is activated (12).

In conclusion, although monoclonal antibodies and decoy receptor inhibiting TNF/TNFR interactions represent pharmacological blockbusters in the treatment of several chronic inflammatory disorders, a better understanding of the ambivalent signals triggered by death receptors is required to foster the clinical development of effective therapeutic molecules.

## Author Contributions

All authors listed have made a substantial, direct and intellectual contribution to the work, and approved it for publication.

## Conflict of Interest

The authors declare that the research was conducted in the absence of any commercial or financial relationships that could be construed as a potential conflict of interest.

## References

[1] Monreal AW Ferguson BM Headon DJ Street SL Overbeek PA Zonana J. Mutations in the human homologue of mouse dl cause autosomal recessive and dominant hypohidrotic ectodermal dysplasia. Nat Genet. (1999) 22:366–9. 10.1038/11937 10431241

[2] Headon DJ Overbeek PA. Involvement of a novel Tnf receptor homologue in hair follicle induction. Nat Genet. (1999) 22:370–4. 10.1038/11943 10431242

[3] Dostert C Grusdat M Letellier E Brenner D. The TNF Family of Ligands and Receptors: communication Modules in the Immune System and Beyond. Physiol Rev. (2019) 99:115–60. 10.1152/physrev.00045.2017 30354964

[4] Dondelinger Y Darding M Bertrand MJ Walczak H. Poly-ubiquitination in TNFR1-mediated necroptosis. Cell Mol Life Sci. (2016) 73:2165–76. 10.1007/s00018-016-2191-4 27066894 PMC4887548

[5] Sato T Irie S Kitada S Reed JC. FAP-1: a protein tyrosine phosphatase that associates with Fas. Science. (1995) 268:411–5. 7536343 10.1126/science.7536343

[6] Fischer R Kontermann RE Pfizenmaier K. Selective targeting of TNF receptors as a novel therapeutic approach. Front Cell Dev Biol. (2020) 8:401. 10.3389/fcell.2020.00401 32528961 PMC7264106

[7] Poissonnier A Sanséau D Le Gallo M Malleter M Levoin N Viel R. CD95-mediated calcium signaling promotes T helper 17 trafficking to inflamed organs in lupus-prone mice. Immunity. (2016) 45:209–23. 10.1016/j.immuni.2016.06.028 27438772 PMC4961226

[8] Edmond V Dufour F Poiroux G Shoji K Malleter M Fouqué A. Downregulation of ceramide synthase-6 during epithelial-to-mesenchymal transition reduces plasma membrane fluidity and cancer cell motility. Oncogene. (2015) 34:996–1005. 10.1038/onc.2014.55 24632610

[9] Qadir AS Ceppi P Brockway S Law C Mu L Khodarev NN. CD95/Fas increases stemness in cancer cells by inducing a STAT1-dependent type I interferon response. Cell Rep. (2017) 18:2373–86. 10.1016/j.celrep.2017.02.037 28273453 PMC5474321

[10] Malireddi RKS Gurung P Kesavardhana S Samir P Burton A Mummareddy H. Innate immune priming in the absence of TAK1 drives RIPK1 kinase activity-independent pyroptosis, apoptosis, necroptosis, and inflammatory disease. J Exp Med. (2020) 217. 10.1084/jem.20191644. [Epub ahead of print]. 31869420 PMC7062518

[11] Green DR Ferguson T Zitvogel L Kroemer G. Immunogenic and tolerogenic cell death. Nat Rev Immunol. (2009) 9:353–63. 10.1038/nri2545 19365408 PMC2818721

[12] Galluzzi L Buque A Kepp O Zitvogel L Kroemer G. Immunogenic cell death in cancer and infectious disease. Nat Rev Immunol. (2017) 17:97–111. 10.1038/nri.2016.107 27748397

